# Data from a comparative proteomic analysis of tumor-derived lung-cancer CD105^+^ endothelial cells

**DOI:** 10.1016/j.dib.2016.03.062

**Published:** 2016-03-22

**Authors:** Hongwei Jin, Xiao Cheng, Yihua Pei, Jianguo Fu, Zhi Lyu, Huifang Peng, Qin Yao, Yu Jiang, Lianzhong Luo, Huiqin Zhuo

**Affiliations:** a Xiamen Center of Clinical Laboratory, The Affiliated Zhongshan Hospital, Xiamen University, Xiamen, Fujian 361004, China; b Respiratory Department, The Affiliated Zhongshan Hospital, Xiamen University, Xiamen, Fujian 361004, China; c Central Laboratory, The Affiliated Zhongshan Hospital, Xiamen University, Xiamen, Fujian 361004, China; d Hospital Infection Control Office, The Affiliated Zhongshan Hospital, Xiamen University, Xiamen, Fujian 361004, China; e State Key Laboratory of Stress Cell Biology, School of Life Science, Xiamen University, Xiamen, Fujian 361004, China; f Central Laboratory, Xiamen Women׳s and Children׳s Hospital, Xiamen, Fujian 361004, China; g Department of Pharmacy, Xiamen Medical College, Xiamen, Fujian 361004, China

**Keywords:** Tumor-derived endothelial cells, Lung cancer, 2D-PAGE, MALDI-TOF, MS/MS

## Abstract

Increasing evidence indicates that tumor-derived endothelial cells (TECs) are more relevant for the study of tumor angiogenesis and for screening antiangiogenic drugs than normal ECs (NECs). In this data article, high-purity (>98%) primary CD105^+^ NECs and TECs purified from a mouse Lewis lung carcinoma model bearing 0.5 cm tumors were identified using 2D-PAGE and Matrix-assisted laser desorption/ionization tandem mass spectrometry (MALDI-MS/MS). All the identified proteins were categorized functionally by Gene Ontology (GO) analysis, and gene-pathway annotated by Kyoto Encyclopedia of Genes and Genomes (KEGG). Finally, protein–protein interaction networks were also built. The proteomics and bioinformatics data presented here provide novel insights into the molecular characteristics and the early modulation of the TEC proteome in the tumor microenvironment.

**Specifications Table**

**Table:** 

Subject area	*Biology*
More specific subject area	*Tumor microenvironment*
Type of data	*Table, figure*
How data was acquired	*Mass spectroscopy, data acquired using* 4800 *Plus MALDI TOF/TOF™ Analyzer*
Data format	*Analyzed*
Experimental factors	*Primary CD105*^*+*^*NECs and TECs were isolated and purified from a mouse Lewis lung carcinoma model bearing* 0.5 cm *tumors*
Experimental features	*The proteins were separated using 2D-PAGE, in-gel digested and analyzed using MALDI TOF/TOF*
Data source location	*Xiamen, Fujian, China*
Data accessibility	*The data is available with this article*

**Value of the data**

• Highly optimized method for primary ECs proteomic analysis by 2D-PAGE from tumor tissues.
• Bioinformatics data can be useful for clarified the heterogeneity of tumor derived ECs.
• The differentially expressed proteins indicate the potential function of the TEC in tumor microenvironment.

## 1 Data

The data is related to the identification and verification of transgelin-2 as a potential biomarker of tumor-derived lung-cancer endothelial cells by comparative proteomics [1]. A highly optimized method for primary CD105^+^ NECs and TECs proteomic analysis by 2D-PAGE and MALDI-MS/MS was presented here. All the identified proteins were categorized by GO, KEGG and protein–protein interaction analysis, to clarify the function of TEC in tumor microenvironment.

## 2 Experiment design, materials and methods

Primary CD105^+^ NECs and TECs were isolated from a mouse Lewis lung carcinoma model bearing 0.5 cm tumors. Differentially expressed proteins were identified using 2D-PAGE and Matrix-assisted laser desorption/ionization tandem mass spectrometry (MALDI-MS/MS). 2D-PAGE was performed using the GE Ettan™ IPGphor™ 3 and DALTsix system. Proteins were visualized by silver staining, and images were recorded on a GE ImageScanner III system and analyzed with the ImageMaster 2D Platinum software. Mass spectrometry data were obtained in an automated analysis loop using a 4800 Plus MALDI TOF/TOF™ Analyzer (Applied Biosystems, USA), and collected using the 4000 Series Explorer™ software and submitted to database search via GPS Explorer™ (Applied Biosystems). MASCOT Server version 2.2 and NCBI non-redundant database were used for protein identification. A total of 63 spots resulted in the identification of 48 unique proteins (28 up- and 20 down-regulated proteins) were detected by at least 1.5-fold changes in TECs. All the identified proteins were categorized functionally by Gene Ontology (GO) analysis. Gene-pathway annotations were compiled from Kyoto Encyclopedia of Genes and Genomes (KEGG), BioCarta, BioCyc, and Reactome. Protein–protein interaction networks were built using the DIP, MINI, BioGRID, IntAct, and STRING databases, and the data were imported into Cytoscape in order to visualize the graphs.

## 3 Establishment of ECs cultures

**Fig. 1:**
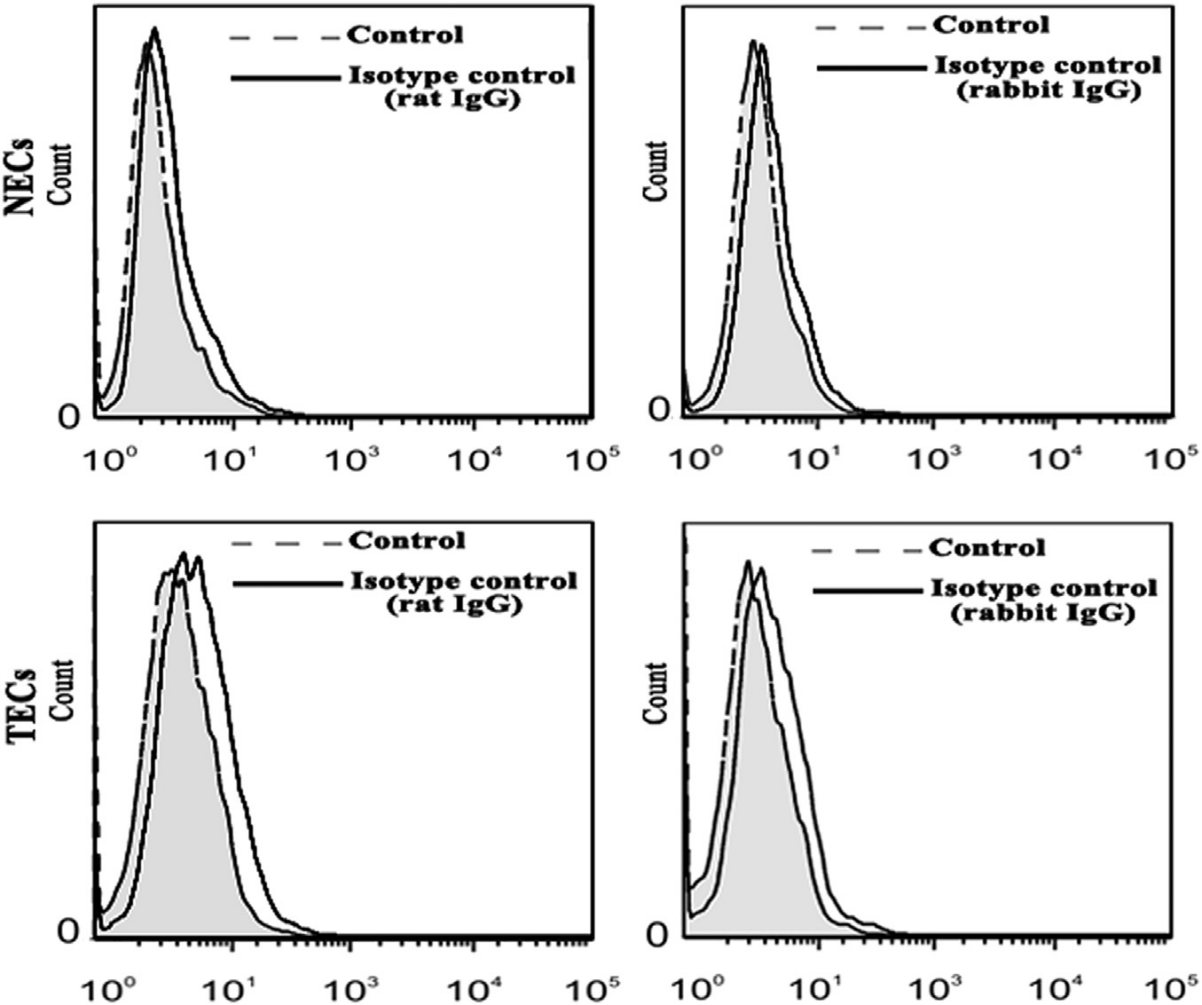
Non-specific staining of endothelial cells was evaluated using isotype controls (rat IgG or rabbit IgG) and matched Alexa Fluor® 555-conjugated secondary antibodies by flow cytometry.

Primary ECs were purified by combining the enzymatic digestion, differential adherence and magnetic cell-sorting using a CD105 MultiSort Kit, according to the procedure described in the Journal of Proteomics paper [1]. Endothelial phenotype and purity were confirmed by cytofluorimetric analysis on the basis of positive expression of a panel of endothelial markers [2], [3], and isotype control stainings were shown in Fig. 1. ECs (CD105 expression of >98%) at first passage were used for the proteomic analysis to maintain the most properties of the in vivo state [4].

## 4 Comparative proteomic analysis of NECs and TECs

NECs and TECs were harvested and suspended in lysis buffer containing 7 M urea, 4% CHAPS, 2 M thiourea, 60 mM DTT, 10 mM Tris, 1 mM EDTA, 0.002% bromophenol blue, and 2% ampholine (pH 3–10) [5]. Cells were disrupted on ice by five 15 s pulses of sonication, followed by five cycles of freeze-thaw: 5 min in liquid nitrogen, 1 min in a 37 °C water bath and 3 min at room temperature. Then, supernatant fractions were collected after centrifugation at 14,000×*g* for 40 min at 4 °C and then stored at −80 °C. Protein concentration was determined using a Bradford assay kit. A non-linear pH gradient of 3–10 was chosen for isoelectric focusing (IEF). The second-dimension was performed on a 12.5% SDS-PAGE to optimize the separation of proteins from 12 to 97 kDa. Before IEF, a solution containing 50 mM MgCl_2_, 1 mg/mL DNase 1, and 0.25 mg/mL RNase A was added to the protein samples at ratio of 1:20 (V/V). Aliquots containing 100 μg of protein were resuspended in 250 μL of rehydration solution. Equal amount of sample was loaded in triplicate. After 18 h of rehydration of the IPG strips (GE Healthcare, USA), IEF was performed using the GE Ettan™ IPGphor™ 3 system at 67,860 V·h. After focusing, the strips were first equilibrated for 15 min in a buffer containing 6 M urea, 20% glycerol, 2%SDS, 2% DTT, and then for 15 min in the same buffer containing 2.5% iodoacetamide instead of DTT. SDS-PAGE was performed on a GE Ettan™ DALTsix system. Finally, the proteins were visualized by silver staining. Briefly, gels were soaked in fix solution (50% ethanol, 10% acetic acid) for at least 45 min, rinsed in 30% ethanol and ddw for 3×10 min, respectively. To sensitize, gels were soaked in sensitivity enhancing solution (2 mL of 10% sodium thiosulfate solution per liter) for 2 min (one gel at a time), followed by rinsed in ddw for 2×1 min. For silver reaction, submerged gel in 0.1% silver stain solution [0.1% silver nitrate with 0.08% formalin (37%)] for 20 min, followed by rinsed in ddw for 2×1 min. Developed image in development solution [2% sodium carbonate with 0.04% formalin (37%)] until desired intensity of staining occur, then quickly washed in 5% acetic acid for 10 min, and rinsed in ddw for 5 min to stop the staining. Finally, all gels were rinsed with water (several changes) prior to drying or densitometry.

**Fig. 2:**
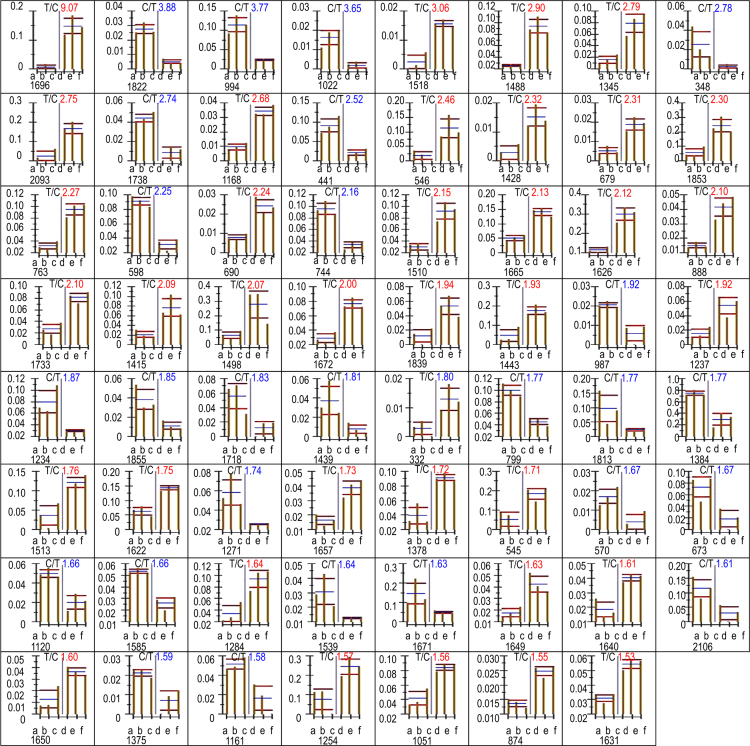
Relative levels of signal intensity in TECs and NECs.

Images were recorded on a GE ImageScanner III system. The gels were analyzed with the ImageMaster 2D Platinum software, and automatic spot matching in conjunction with detailed manual checking of the spot finding, to identify proteins in both the NECs and TECs. The quality of the gels was verified by using the quality control of the software. Spot intensities were expressed as the percentage of the integrated spot density (volume) over the total density of all measured spots. Significantly over-abundant spots were detected at a significance level of 5% and a fold number of >1.5. After statistical analysis, 63 spots were identified in TECs, compared with NECs, and the histograms in Fig. 2 show the relative levels of signal intensity. The histograms contain information about spot ID, spot intensity, relative ratio, and statistical result of triplicate repeats. Spots that were differentially expressed between NECs and TECs were then isolated and identified using mass spectrometry as described below.

Gels were analyzed with the ImageMaster 2D Platinum software. The quality of the gels was verified by using the quality control of the software. Spot intensities were expressed as the percentage of the integrated spot density (volume) over the total density of all measured spots. Significantly over-abundant spots were detected at a significance level of 5% (*p*-value < 0.05%) and a fold number of >1.5.

Differentially expressed protein spots were picked manually and enzymatic digestion in-gel was carried out according to the procedure of Zimmerman et al. with some modifications [6]. Briefly, dried gel pieces were incubated with 10 μL of 25 μg/mL sequencing-grade trypsin (Promega) in 40 mM ammonium bicarbonate for 30 min at 4 °C. Then another 20 μL of 40 mM ammonium bicarbonate was added to ensure complete cover of the pieces. Digestion was carried out at 37 °C for 12 h and peptides were recovered by sequencing extractions with 25 mM ammonium bicarbonate, 50% ACN/0.1% TFA, and 100% ACN, and all steps were repeated once more.

## 5 Database searching

MALDI-MS/MS data were obtained in an automated analysis loop using a 4800 Plus MALDI TOF/TOF™ Analyzer (Applied Biosystems, USA). Digested peptides were desalted using C18 ZipTips® (Millipore, USA). MS and MS/MS spectra were collected using the 4000 Series Explorer™ software and submitted to database search via GPS Explorer™ (Applied Biosystems). MASCOT Server version 2.2 (Matrix Science, London, UK) and the NCBI non-redundant database were used for protein identification. The search parameters were set as follows: taxonomy: Mouse; mass values, monoisotopic; precursor mass tolerance, ±1 Da; fragment mass tolerance, ±0.3 Da; enzyme, trypsin; maximum missed cleavage allowed, 1; modifications, carbamidomethyl Cys (permanent); methionine oxidation (variable); Ser, Thr, and Tyr phosphorylation. Results were scored using the probability-based MASCOT score. Among these identified proteins, many have been identified in previous cancer studies, including Tagln2, Hspd1, Pgam1, Dld, Cct2, Npm1, Arhgdia, Gdi2, Aprt, Park7, Acy1, Capzb, Ctsb, Hnrnpk, Vcp, Enol, Pkm2, Pcna, Pgk1, and Map2k1, which are list in Table 1. For these candidate biomarkers, our results are in agreement with published data.

## 6 Real-time PCR analysis of selected proteins

**Fig. 3:**
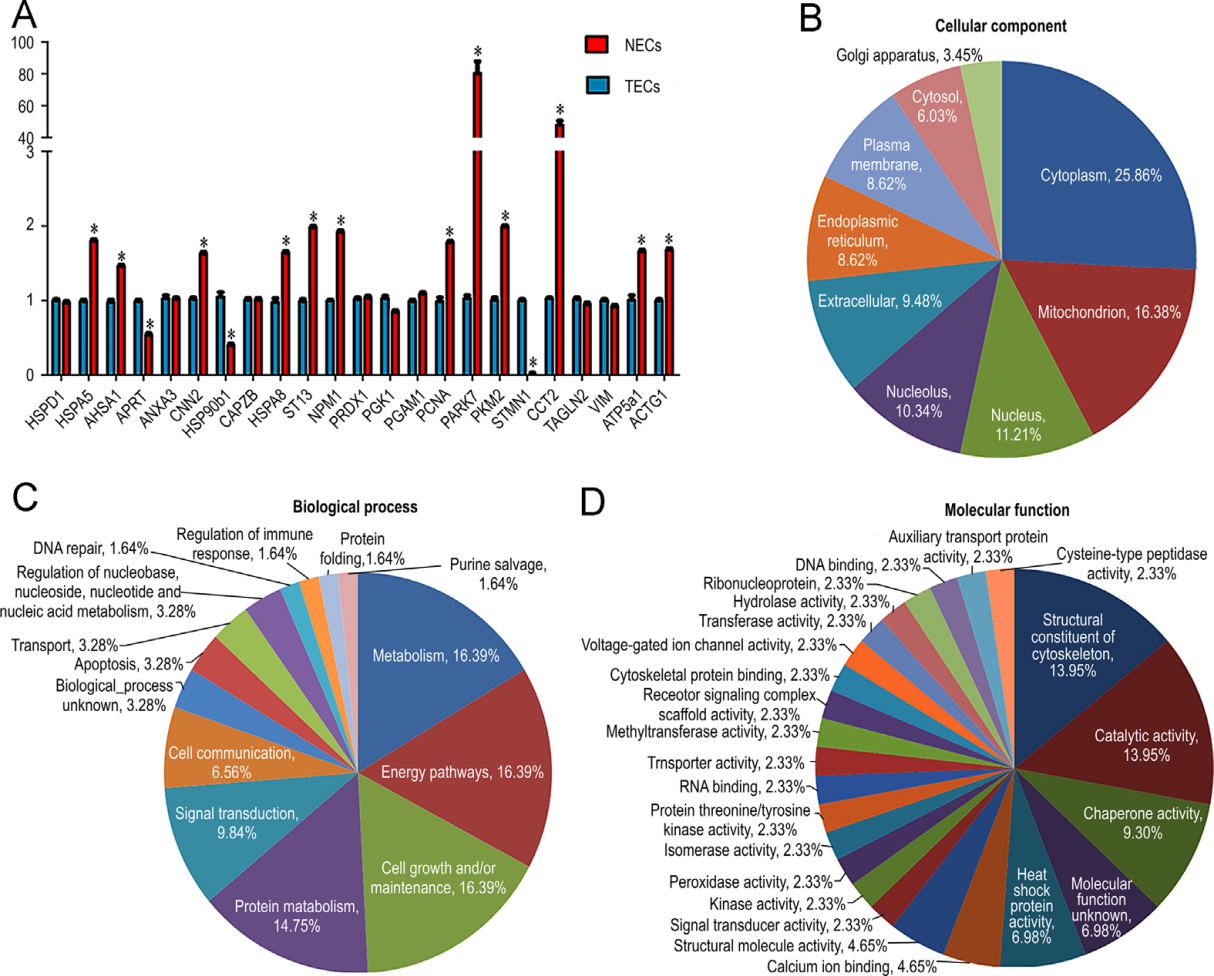
Verification of differentially expressed proteins by real-time RT-PCR of 23 selected transcripts (A) and analysis of functional distribution of proteomically identified endothelial proteins involved in cellular components (B), biological processes (C), and molecular function (D) (based on the Human Protein Reference Database).

On the basis of GO annotations (20 proteins, including in the top 10 GO BP terms) and protein–protein interaction analysis results (3 proteins), the mRNA levels of 23 differentially expressed proteins were analyzed by real-time RT-PCR. Total RNA was extracted using TRIzol. RT-PCR analysis was performed by using the SYBR^®^ Green I RT-PCR Master Mix kit from Bio-Rad Laboratories, Inc. on a Rotor-Gene 3000 system. The relative mRNA levels of differentially expressed proteins were normalized to that of *GAPDH*, and NECs were used for calibration. Primers for selected proteins are listed in Table 2. Measurement of △Ct was performed in triplicate. RT-PCR data were analyzed for relative gene expression using the △△Ct method. The results of the RT-PCR analysis were mostly consistent with those obtained in the 2D-PAGE analysis (see Fig. 3A).

## 7 Bioinformatics analysis of the identified proteins

To get a precise prediction, multiple bioinformatics methods were performed. First, the mouse genes thus identified were associated with their putative human orthologs using NCBI׳s HomoloGene resource. Homogene annotations were downloaded from “ftp://ftp.ncbi.nih.gov/pub/HomoloGene/build67/homologene.data.” Then, the molecular functions of the all identified proteins were assigned on the basis of a search against the Human Protein Reference Database (HPRD, HPRD_Release9_041310.tar.gz). Results including biological process, cellular component, and molecular function were shown in Fig. 3B–D.

**Fig. 4:**
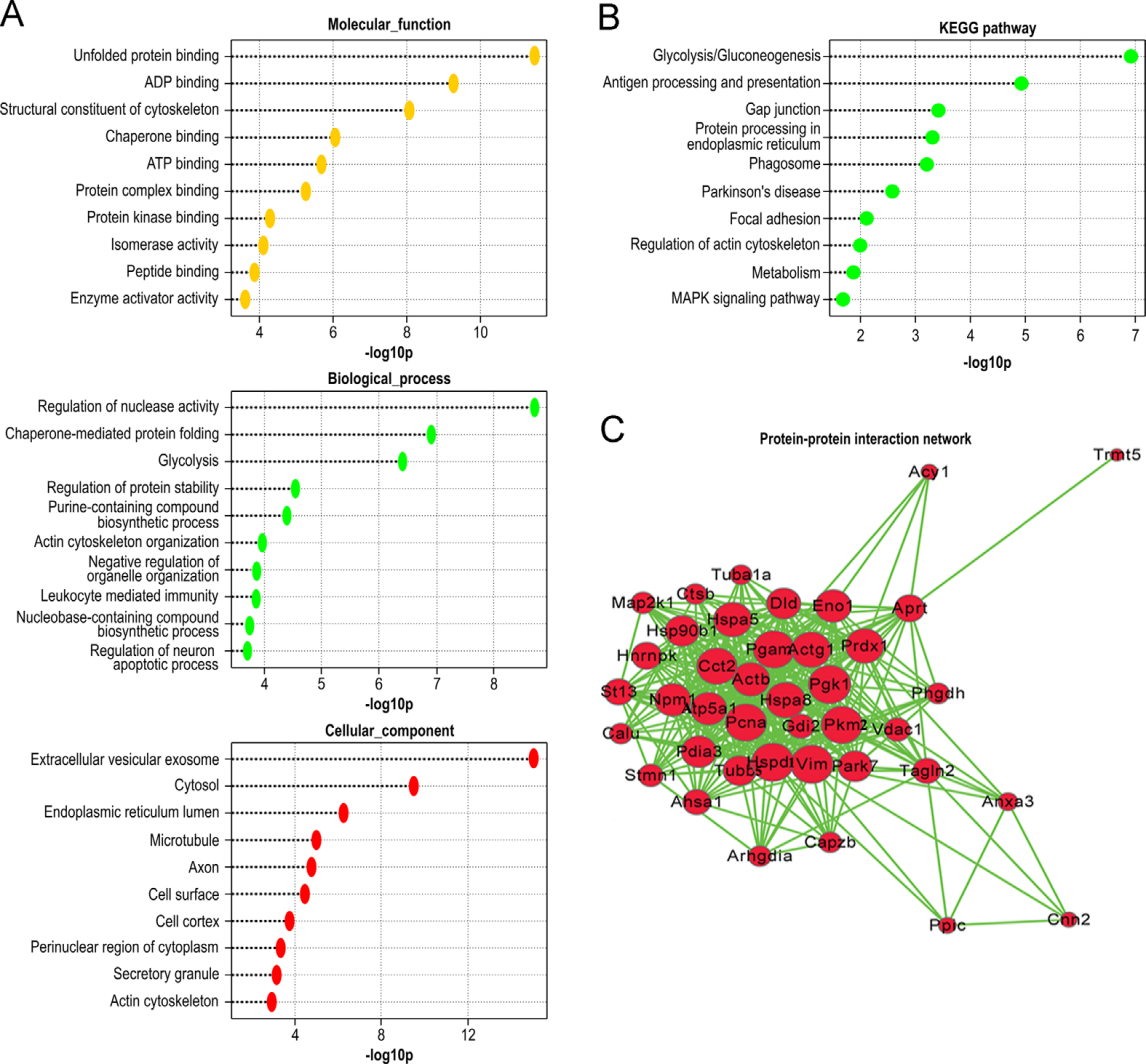
List of the top 10 gene ontology (GO; A), Kyoto Encyclopedia of Genes and Genomes (KEGG) pathways (B) and protein–protein interaction networks for differentially expressed proteins in TECs. Proteins were uploaded into the Ingenuity Pathway analysis (IPA) software server. The network was built using the STRING (http://string-db.org/) database, and the data were imported into Cytoscape (www.cytoscape.org) for visualization.

Second, all the identified proteins were categorized functionally by GO analysis. GO was downloaded from the GeneOntology website (geneontology.org/ontology/ geneontology_edit.obo). Corresponding mouse GO-gene annotations were downloaded from the NCBI Entrez Gene ftp website (ftp://ftp.ncbi.nih.gov/gene/DATA/gene2go.gz). The GO analysis results, including the biological process (BP), cellular component (CC), and molecular function (MF), were generated. Gene set enrichment analysis revealed that all the differentially expressed proteins were enriched in 99 GO terms (*p*<0.05), including 58 BP, 23 MF and 18 CC. The top 10 GO terms ranked according to their significance level were listed in Fig. 4A.

Third, gene-pathway annotations were compiled from Kyoto Encyclopedia of Genes and Genomes (KEGG), BioCarta (http://www.biocarta.com/), BioCyc, and Reactome. A hypergeometric test was chosen for statistical analysis, and significantly enriched pathways were identified at a corrected *p*-value of <0.05. Results were listed in Fig. 4B.

Forth, protein–protein interaction networks were built using the Database of Interacting Proteins (DIP), Molecular Interaction (MINI), Database of Protein and Genetic Interaction (BioGRID), IntAct molecular interaction (IntAct), and STRING (http://string-db.org/) databases, and the data were imported into Cytoscape in order to visualize the graphs. The graphs was shown in Fig. 4C, and the details of top 10 proteins were listed in Table 3, including the degree, betweenness, gene ontology, and KEGG pathway.

## 8 Verification of candidate proteins in clinical samples

Lung squamous cell carcinoma specimens from 30 patients (11 lung squamous cell carcinoma and 19 adenocarcinoma) were chosen for IHC analysis. Histopathology reports were also obtained along with the samples, and shown in Table 4. Serum samples from 54 LC patients, 31 colorectal cancer patients, 31 esophageal cancer patients, and 84 normal individuals were used for the ELISA analysis. The clinical data of the LC patients are presented in Table 5.

## Figures and Tables

**Fig. S1:**
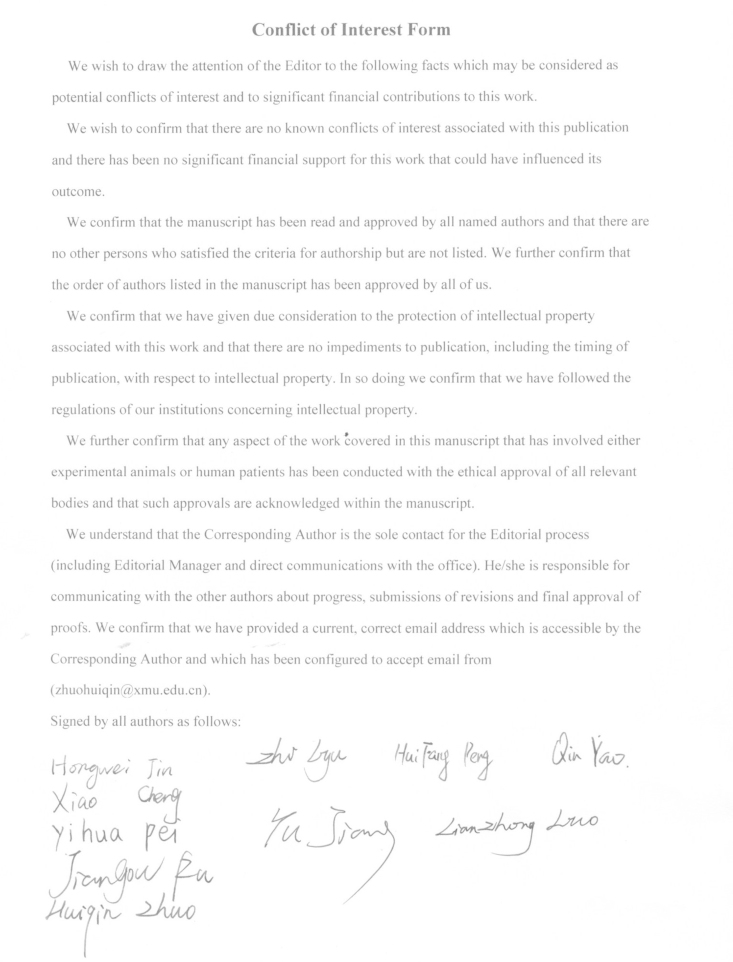


**Table 1:** Cancer proteomic identifications used for comparison.

**Symbol**	**Protein identified**	**Reference**
Tagln2	Transgelin-2	[7], [8]
Hspd1	Heat shock protein 1	[9], [10], [11]
Pgam1	Phosphoglycerate mutase 1	[9], [12]
Dld	Dihydrolipoyl dehydrogenase	[13]
Cct2	Chaperonin containing Tcp1, subunit 2 (beta)	[14], [15]
Npm1	Nucleophosmin	[7]
Arhgdia	Rho GDP dissociation inhibitor (GDI) alpha	[16]
Gdi2	Rab GDP dissociation inhibitor beta	[8]
Aprt	Adenine phosphoribosyl transferase	[17]
Park7	Parkinson disease7	[18]
Acy1	Aminoacylase-1	[10]
Capzb	Capping protein (actin filament) muscle Z-line, beta	[7]
Ctsb	Cathepsin B	[19]
Hnrnpk	Heterogeneous nuclear ribonucleoprotein K	[15]
Vcp	Transitional endoplasmic reticulum ATPase	[20]
Enol	Enolase 1, alpha non-neuron	[10], [12], [21], [22]
Pkm2	Pyruvate kinase isozymes M2	[23]
Pcna	Proliferating cell nuclear antigen	[3], [24]
Pgk1	Phosphoglycerate kinase 1	[25]
Map2k1	Mitogen-activated protein kinase kinase 1	[26]

**Table 2:** Primers for real-time RT-PCR amplification.

No.	Gene Symbol	Primer sequence (5′ to 3′)	
1	*GAPDH*	Forward: ctgcgacttcaacagcaact	Reverse: gagttgggatagggcctctc
2	*HSPD1*	Forward: tagctgttacaatggggcca	Reverse: ggcaacgtcctgaacaagtt
3	*HSPA5*	Forward: ccccaactggtgaagaggat	Reverse: ccccaagacatgtgagcaac
4	*AHSA1*	Forward: tcaccggggagtttactgac	Reverse: tcaaagtagtaccgctgcca
5	*APRT*	Forward: agcgtgctgatacctacctc	Reverse: aggagtccgggtctttcaag
6	*ANXA3*	Forward: tcaagcaggcagatgaagga	Reverse: tggccagatgttcatccact
7	*CNN2*	Forward: tctatgcagaactggcacca	Reverse: gcgtcgtcaaagttcctctc
8	*HSP90B1*	Forward: agtcgggaagcaacagagaa	Reverse: tctccatgttgccagaccat
9	*CAPZB*	Forward: gccgtactgcccattacaag	Reverse: atgttggctatgtgtgggga
10	*HSPA8*	Forward: tctaagggacctgcagttgg	Reverse: ttgcaacctgattcttggcc
11	*ST13*	Forward: aggaagcagctcatgacctt	Reverse: tcgagccttcttcacccttt
12	*NPM1*	Forward: tgtttccggatgactgacca	Reverse: cttggcaagtgaacctggac
13	*PRDX1*	Forward: aagagcaacggggttcctaa	Reverse: ggccagcctagtctacagag
14	*PGK1*	Forward: gatgcttttgggactgcaca	Reverse: tcagctggatcttgtctgca
15	*PGAM1*	Forward: catcatggagctgaacctgc	Reverse: tcgccttcacttcttcacct
16	*PCNA*	Forward: gtggagcaacttggaatccc	Reverse: ggttaccgcctcctcttctt
17	*PARK7*	Forward: ggagcagaggagatggagac	Reverse: tctgtgcacccagatttcct
18	*PKM1*	Forward: ctggaatgaatgtggctcgg	Reverse: taagcgttgtccagggtgat
19	*STMN1*	Forward: attctcagccctcggtcaaa	Reverse: gagctgcttcaagacttccg
20	*CCT2*	Forward: cgctgtggatcatggttctg	Reverse: gccagactcccacctagttt
21	*TAGLN2*	Forward: ctcttcgatggccttcaagc	Reverse: cgagaagttccgagggttct
22	*VIM*	Forward: cgctttgccaactacatcga	Reverse: cctcctgcaatttctctcgc
23	*ATP5α1*	Forward: gagagcagccaagatgaacg	Reverse: gacacgggacacagacaaac
24	*ACTG1*	Forward: ccctatcgaacacggcattg	Reverse: cctgaatggccacgtacatg

**Table 3:** The top 10 differentially expressed proteins sorted by network betweeness.

**Protein**	**Degree**	**Betweenness**	**Gene Ontology**	**KEGG Pathway**
Vim	29	0.0828	(GO:1900147) regulation of Schwann cell migration; (GO:0045103) intermediate filament-based process	
Aprt	17	0.0601	(GO:0006166) purine ribonucleoside salvage; (GO:0006168) adenine salvage	(00230) Purine metabolism; (01100) Metabolism
Hspd1	29	0.0490	(GO:0002842) positive regulation of T cell mediated immune response to tumor cell; (GO:0043065) positive regulation of apoptotic process; (GO:0043066) negative regulation of apoptotic process	(03018) RNA degradation; (04940) Type I diabetes mellitus
Pgam1	28	0.0398	(GO:0006096) glycolysis; (GO:0006110) regulation of glycolysis; (GO:0008152) metabolic process; (GO:0043456) regulation of pentose- phosphate shunt; (GO:0045730) respiratory burst	(00010) Glycolysis/Gluconeogenesis; (01100) Metabolism
Pkm2	28	0.0375	(GO:0001889) liver development; (GO:0008152) metabolic process; (GO:0031100) organ regeneration;	(00010) Glycolysis/Gluconeogenesis; (00230) Purine metabolism;(00620) Pyruvate metabolism;(01100) Metabolism;(04930) Type II diabetes mellitus
Pgk1	29	0.0353	(GO:0005975) carbohydrate metabolic process;(GO:0006094) gluconeogenesis;(GO:0006096) glycolysis;(GO:0016310) phosphorylation	(00010) Glycolysis/ Gluconeogenesis; (01100) Metabolism
Prdx1	25	0.0306	(GO:0008283) cell proliferation; (GO:0019430) removal of superoxide radicals; (GO:0034101) erythrocyte homeostasis	(04146) Peroxisome
Pcna	28	0.0285	(GO:0000122) negative regulation of transcription from RNA polymerase II promoter;(GO:0006260) DNA replication	(03030) DNA replication;(03410) Base excision repair;(03420) Nucleotide excision repair;(03430) Mismatch repair;(04110) Cell cycle
Cct2	27	0.0277	(GO:0006457) protein folding; (GO:0007339) binding of sperm to zona pellucida;(GO:0044267) cellular protein metabolic process; (GO:0051131) chaperone-mediated protein complex assembly	
Tagln2	15	0.0248	(GO:0007517) muscle organ development	

**Table 4:** Clinical data of lung cancer patients used in the immunohistochemical analysis.

No.	Gender	Age (years)	History of smoking	Histological grade	Lymph node metastasis	Tumor size	Type 1	Type 2	TMN staging
1	M	47	Y	M	N	5.2×2.4×2.0	A	P	T2N2M0
2	M	29	N	H	N	0.6×0.5×0.4	A	C	T1NOM0
3	M	62	Y	M	N	9.5×8.4×4.7	S	C	T3N0M0
4	M	56	N	H	N	2.3×2.7×1.6	A	P	T2N0M0
5	M	56	Y	L	Y	2.1×1.5×1.3	A	P	T4N2M1
6	M	56	Y	M	N	7.0×6.5×5.0	S	C	T2N2M0
7	M	54	Y	H	Y	3.0×1.5×0.7	S	C	T2N3M0
8	M	70	Y	M	N	7.5×7.3×6.0	S	C	T3N0M0
9	M	59	Y	M	N	1.8×1.8×0.7	A	P	T2N0M0
10	F	61	N	M	N	1.8×1.6×1.2	A	P	T2N0M0
11	M	66	Y	M	N	1.5×1.0×0.6	S	P	T1N0M0
12	F	62	N	L	Y	5.5×3.5×2.5	S	P	T2N2M0
13	M	59	Y	M	Y	6.5×6.0×5.0	S	C	T4N2M0
14	M	65	Y	M	N	3.8×2.4×2.0	S	C	T2N0M0
15	F	47	N	M	N	2.5×1.5×1.4	A	P	T2N0M0
16	F	62	N	M	Y	2.7×2.7×1.6	A	P	T2N2M0
17	M	68	Y	M	N	8.5×5.8×5.1	S	C	T3N0M0
18	M	69	Y	L	Y	2.2×2.2×1.2	A	P	T2N2M0
19	M	64	Y	M	N	3.5×2.5×0.8	S	C	T2N0M0
20	M	68	Y	L	N	8.0×8.0×7.0	A	P	T3N0M0
21	M	66	Y	M	N	5.5×5.0×4.0	A	P	T2N0M0
22	M	49	Y	L	Y	1.5×1.4×1.4	A	P	T1N1M0
23	M	65	Y	M	N	1.3×1.0×0.7	P	A	T1N0M0
24	F	49	N	M	N	1.7×1.4×1.4	A	P	T2N0M0
25	M	55	Y	M	N	4.5×4.0×3.0	A	P	T2N0M0
26	M	57	Y	M	N	0.5×0.5×0.4	S	C	T1N0M0
27	M	48	Y	H	N	0.7×0.5×0.3	A	C	T1N0M0
28	M	56	Y	M	N	2.0×1.5×1.3	A	P	T2N0M0
29	F	51	N	M	N	1.6×1.4×1.4	A	P	T1N0M0
30	M	78	N	M	N	2.6×2.2×1.7	A	P	T2N0M0

Note: Gender: M=male, F=female; History of smoking: Y=yes, N=no; Histological grade: H=highly differentiated, M=moderately differentiated, L=low differentiation; Lymph node metastasis: Y=yes, N=no; Type 1: S=squamous cell carcinoma, A=adenocarcinoma; Type 2: P=peripheral type, C=central type.

**Table 5:** Clinical data of lung cancer patients used in the ELISA analysis.

No.	Gender	Age (years)	History of smoking	Histological grade	Lymph node metastasis	Tumor size	Stage	Type 1	Type 2	Visceral pleura metastasis	Macrovascular invasion	Nerve infiltration
1	M	61	N	M	N	3.0×1.3×1.0	I	A	C	Y	N	N
2	M	73	N	M	N	12.0×10.0×5.0	II	S	P	N	N	N
3	M	57	Y	M	Y	2.5×2.5×2.0	III	A	P	Y	N	N
4	F	57	N	L	Y	1.2×1.0×0.6	III	S	P	N	N	N
5	F	61	N	M	N	2.0×1.8×1.5	III	A	P	N	Y	N
6	M	49	Y	M	N	3.5×2.8×2.0	I	S	P	N	N	N
7	M	58	Y	L	Y	6.0×5.0×2.0	III	S	C	N	N	Y
8	M	56	Y	M	Y	2.5×2.0×2.0	I	A	P	Y	Y	Y
9	F	51	N	M	Y	3.6×2.6×1.8	III	A	P	Y	N	N
10	M	49	Y	L	N	4.0×3.5×3.0	I	S	P	N	N	N
11	M	66	Y	M	Y	3.0×2.6×2.3	I	S	C	N	Y	Y
12	M	64	Y	M	Y	4.5×4.0×4.0	III	S	P	Y	Y	Y
13	M	68	Y	M	N	8.5×6.8×5.0	II	A	P	Y	N	N
14	M	71	Y	M	Y	7.0×6.0×3.0	III	S	P	Y	Y	N
15	M	72	Y	M	N	2.2×2.0×0.8	I	S	P	Y	N	Y
16	F	71	N	M	Y	7.0×6.0×3.3	III	S	C	N	N	N
17	M	53	Y	L	N	2.5×2.0×2.0	I	A	P	Y	N	N
18	M	52	Y	M	Y	4.0×2.5×2.0	III	S	C	N	Y	Y
19	F	74	N	M	N	1.0×0.3×0.1	I	A	P	N	N	N
20	M	59	Y	L	Y	7.5×5.0×4.0	III	S	C	Y	N	Y
21	M	58	Y	M	Y	2.3×2.0×1.3	I	A	P	N	N	N
22	F	58	N	H	N	2.5×2.5×2.0	I	A	P	Y	N	Y
23	M	58	Y	L	N	4.0×3.0×2.5	I	S	C	N	N	Y
24	M	72	Y	M	Y	5.5×3.5×3.0	III	S	C	N	Y	Y
25	M	61	N	M	Y	5.1×3.2×2.2	III	A	P	Y	Y	Y
26	F	61	N	M	N	1.8×1.6×1.2	I	A	P	Y	N	N
27	M	54	Y	M	Y	3.0×1.5×0.7	III	S	C	N	Y	N
28	M	59	Y	M	N	1.8×1.8×0.7	I	A	P	Y	N	N
29	F	64	N	M	N	2.2×1.7×1.5	I	A	P	Y	N	N
30	M	70	Y	M	N	7.5×7.3×6.0	II	S	P	Y	N	Y
31	M	66	Y	L	N	1.5×1.0×0.6	I	S	P	N	N	N
32	M	79	Y	M	N	3.5×3.3×1.6	I		P	N	N	N
33	M	73	Y	L	N	5.6×4.8×4.2	III	A	P	N	N	Y
34	F	51	N	H	N	1.1×0.7×0.5	I		P	N	N	N
35	M	68	Y	M	N	8.5×5.8×5.1	II	S	C	N	N	Y
36	M	65	Y	M	N	3.8×2.4×2.0	I	S	C	N	N	N
37	M	69	Y	L	Y	2.2×2.2×1.2	III	A	P	Y	Y	N
38	M	64	Y	M	N	3.5×2.5×0.8	I	S	C	N	N	N
39	M	63	Y	M	N	4.6×3.5×3.3	I	S	C	N	N	Y
40	M	54	N	M	Y	2.2×1.9×1.1	II	A	P	N	Y	Y
41	M	58	Y	L	N	6.6×4.4×4.2	II	S	C	N	N	Y
42	F	53	N	L	N	2.2×1.6×1.5	I	A	P	Y	N	Y
43	M	48	Y	M	N	0.7×0.5×0.3	I	A	C	N	N	N
44	M	72	N	M	Y	3.0×2.0×0.8	III	A	C	N	N	N
45	M	58	Y	M	Y	5.0×3.5×3.0	III	S	C	N	Y	Y
46	M	60	Y	M	Y	9.3×8.3×5.3	III	A	P	Y	N	N
47	M	63	Y	L	N	7.0×5.8×5.2	II	A	P	Y	N	N
48	M	59	Y	L	Y	5.0×4.5×4.0	III	S	C	Y	N	Y
49	F	69	N	M	N	2.5×2.0×1.7	I	A	P	N	N	N
50	M	65	Y	M	N	1.3×1.0×0.7	I	A	P	N	N	N
